# Profiling the Functional Diversity of Termite Mound Soil Bacteria as Revealed by Shotgun Sequencing

**DOI:** 10.3390/genes10090637

**Published:** 2019-08-23

**Authors:** Ben Jesuorsemwen Enagbonma, Bukola Rhoda Aremu, Olubukola Oluranti Babalola

**Affiliations:** Food Security and Safety Niche, Faculty of Natural and Agricultural Sciences, North-West University, Private Mail Bag X2046, Mmabatho 2735, South Africa

**Keywords:** metabolic potentials, metagenomics, novel genes, illumina sequencing, termitarium

## Abstract

Profiling the metabolic processes performed by bacteria is vital both for understanding and for manipulating ecosystems for industrial or research purposes. In this study we aim to assess the bacterial functional diversity in termite mound soils with the assumption that significant differences will be observed in the functional diversity of bacteria between the termite mound soils and their surrounding soils and that each environment has a distinguishing metabolic profile. Here, metagenomic DNA extracted from termite mound soils and their corresponding surrounding soils, which are 10 m apart, were sequenced using a shotgun sequencing approach. Our results revealed that the relative abundances of 16 functional categories differed significantly between both habitats. The α diversity analysis indicated no significant difference in bacterial functional categories within the habitats while the β diversity showed that the bacterial functional categories varied significantly between the termite mound soils and the surrounding soil samples. The variations in soil physical and chemical properties existing between the two environments were held accountable for the differences in bacterial functional structure. With the high relative abundance of functional categories with unknown function reported in this study, this could signify the likelihood of getting novel genes from termite mound soils, which are needed for research and commercial applications.

## 1. Introduction

Termite mound soils account for about ten percent of African soils in tropical environments [1]. The termites’ activities during mound construction have a considerable impact on soil morphology such as the formation of subsurface horizons, soil structures, soil aeration, aggregation, and texture [2]. This may in turn have an effect of the bacterial diversity in termite mound soils [1]. Some bacteria isolated from termite mound soils could serve as potential materials for the following: Antimicrobial production [3,4], bioremediation [5], bio-filtering [6], biofertilizers, and biocontrol [7]. The presence of these bacteria may lead to an increase in soil fertility and crop production, health improvement, and environmental sustainability [8].

Harry, et al. [9] have reported that termite mound soils are hotspots for bacteria concentration and high nutrient concentrations were held accountable for it. Termite mound soils have been reported to have high amounts of soil organic matter as well as phosphorus and nitrogen [10]. The physiochemical parameters of termite mounds and their surrounding soils are relatively different [11]. Physical properties and the amount of soil organic matter (SOM) are strong factors that determine microbial diversity [12]. Spain et al. [13] stated that the populations of bacteria are more abundant in termite mound soils than their surrounding soils. This claim was reinforced by a study by Kumar et al. [14], which reported that bacterial populations in closed and open termite mound soils were 65.5 × 10^5^ cfu/g of soil and 75.5 × 10^5^ cfu/g of soil, correspondingly, and they were higher than the surrounding soil with 30.5 × 10^5^ cfu/g of soil bacterial population.

While there is an increase in the knowledge of the compositional and structural diversity of bacteria in termite mound soils, the same cannot be said of the functional diversity. Soil microbial functional diversity is an essential pointer to evaluate ecological processes and functions [15] like mineralization, decomposition, promotion of plant growth, and the release of atmospheric gases like greenhouse gases [16,17]. Therefore, in this study we aim to assess the bacterial functional diversity in termite mound soils. This research hypothesis assumes that significant differences will be observed in the functional diversity of bacteria between the termite mound soils and their surrounding soils and that each environment has a distinguishing metabolic profile. We also assumed that the bacterial functional diversity will be driven by soil physicochemical parameters.

Considering the fact that surrounding soils are more dynamic, heterogeneous, and faced with issues of lower nutrient concentrations than termite mound soils [10,18], bacteria in surrounding soils have to rely on different carbon sources if they are to survive such habitats [19]. Thus, we anticipated that there would be relatively higher abundances of genes relating to carbon sources like the following: (a) carbohydrate metabolism, (b) amino acids and derivatives, (c) fatty acids, lipids, and isoprenoids, and competitive related genes like (d) metabolism of aromatic compounds and (e) motility and chemotaxis in the surrounding soil samples than the termite mound soils.

Termites in the mounds are usually faced with *Metarhizium anisopliae* (a fungal entomopathogen) and they survive due to certain bacteria (like *Streptomyces*) which provide meaningful protection to the colony through various processes driven by the products of core housekeeping genes [20]. Thus, we also anticipated that there would be relatively higher abundances of genes relating to (a) virulence, disease, and defense, (b) genes related to cell division, (c) DNA metabolism, (d) protein metabolism, (e) phages, prophages, and transposable elements in in the termite mound soils than their surrounding soil samples.

To test our hypotheses, the shotgun metagenomic sequencing approach, which is the direct sequencing of the whole genomes found in a given ecological sample, was employed. Metagenomics and high-throughput sequencing applications have made it much more possible to generate genetic information of potential genes from bacteria. They also give a better understanding of the different activities and processes carried out by soil bacteria, irrespective of their environment [21,22].

## 2. Materials and Methods

### 2.1. Study Sites and Soil Sampling

**Figure 1:**
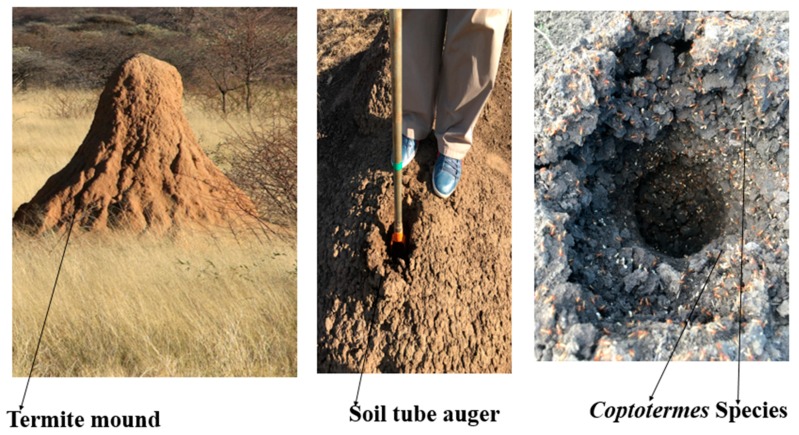
Termite mounds colonized by *Coptotermes* species.

Soil samples of 50 g were collected from 1 m depth using a 5 cm diameter split tube auger from four different termite mounds (about 2 m apart) from Braklaagte (T1) and four different termite mounds (about 2 m apart) from Zeerust (T2). The termite mounds (Figure 1) were colonized by *Coptotermes* species. For comparison purposes, four samples of corresponding surrounding soils from Braklaagte (S1) and four samples of corresponding surrounding soils from Zeerust (S2), which were 10 m apart from the termite mounds were also collected. The soil samples were kept briefly in cooler boxes filled with ice blocks during sampling and transportation and then moved to the laboratory during the same day where they were stored in a fridge at 4°C for 14 days for further analysis (DNA isolation and physicochemical analysis). After soil analysis, the mean values of all 4 samples from each site (T1, T2, S1, and S2) were used for statistical analysis. Both Braklaagte (25°26′13.5″ S 26°05′50.4″ E) and Zeerust (25°27′11.2″ S 26°07′33.8″ E) are in North West Province, South Africa. North West Province is an inland province in South African that borders Botswana. The background is demarcated by mountains in the north-east and it is distributed with shrubs and trees. Its average temperatures vary from 3 to 21°C and 17 to 31°C in winter and summer, respectively. The yearly rainfall of the area is approximately 360 mm, with most falling between October and April (https://www.south-africa-info.co.za/country/article/511/an-overview-of-the-north-west-province).

### 2.2. Soil Analysis of Termite Mound Soils and Their Comparative Surrounding Soil Samples

Soil properties were assessed within 14 days of sampling. The soil samples of 20 g were air dried, ground, mixed well, and passed through a 2 mm sieve to remove rubble and solid wooden materials for soil analysis. Particle size analyses were done via the hydrometer technique [23]. The United States Department of Agriculture (USDA) particle size classes, namely sand (2.0–0.05 mm), silt (0.05–0.002 mm), and clay (<0.002 mm), were followed for assigning textural classes. Soil pH in distilled water were measured using a pH-meter in a 1:2.5 soil:water ratio and the total nitrogen was determined by the Kjeldhal method according to the procedures used by Muwawa et al. [24]. Exchangeable calcium (Ca), magnesium (Mg), and potassium (K) were analyzed after extraction using 1M ammonium acetate method at pH 7.0. Exchangeable Ca and Mg in the extracts were read using an atomic absorption spectrophotometer (AAS), whereas exchangeable K was read by a flame photometer [11]. Available phosphorus (P) was determined spectrophotometrically while organic carbon was determined using the dichromate digestion [25].

### 2.3. Metagenomic DNA Extraction and Sequencing

A PowerSoil DNA isolation kit (MoBio Laboratories, Inc., Carlsbad, CA, USA) was used to extract whole microbial DNA from 0.25 g each of the soil samples collected from the termite mounds and their corresponding surrounding soils, following the manufacturer’s procedure. All datasets were produced by whole-metagenome shotgun sequencing at Molecular Research LP (MR DNA, Shallowater, TX, USA). The concentration of the DNA was measured by fluorescence using the Quant-iT PicoGreen dsDNA kit (Invitrogen, Carlsbad, CA, USA). Fluorescence was assessed on a DQ 300 fluorometer (Hoefer Scientific Instruments, San Francisco, CA, USA). A total of 50 ng of DNA from each sample was used to prepare the libraries using a Nextera DNA Sample Preparation Kit (Illumina). Library insert size was determined by an Experion Automated Electrophoresis Station (Bio-Rad). The insert size of the libraries ranged from 300 to 850 bp (average 500 bp). Each library was loaded to a 600 Cycles v3 Reagent cartridge (Illumina) and the sequencing was performed using a 2 × 250 base pair sequencing run on the Illumina MiSeq 2500 platforms (San Diego, CA, USA).

### 2.4. Metagenome Annotation And Data Analysis

The raw sequences of each of the metagenomes were uploaded to the metagenomics rapid annotation online server (MG-RAST) at http://www.mg-rast.org [26]. In the MG-RAST server, the sequences were subjected to quality control. This comprises of dereplication—that is the removal of artificial sequences formed by sequencing artifacts, removing host specific species sequences, ambiguous base filtering (removing sequences with >5 ambiguous base pairs with 15 phred score cutoff) and a length filtering (removing sequences with a length of >2 standard deviation from the mean). Following quality control (QC), sequences were annotated using the BLAT (the BLAST-like alignment tool) algorithm [27] against the M5NR database [28], which provides nonredundant integration of many databases. Bacterial classifications were performed by the SEED Subsystem (result shown in supplementary Figure S1) and, also, functional categories assignments were performed by the SEED Subsystems level 1, level 2, and level 3 databases. An e-value of 1e–5, a minimum identity of 60%, and a maximum alignment length of 15 base pairs were the conditions used when the bacteria classifications and functional gene categories were assigned. No further analyses were carried out on sequences that failed annotation. Our focus was on bacteria, which were approximately 99% of the entire sequences. Hence, we then discarded sequences obtained from viruses, archaea, and eukaryotes. To decrease the effect of experimental noise/error, the normalized data option of MG- RAST was applied. The resulting functional table was agglomerated accordingly to each functional level and unclassified reads were retained for statistical purposes. Next, the abundances were transformed into percentages. After the 16 sequences were analyzed individually with MG-RAST, the mean values of the relative abundances of all 4 samples from each site (T1, T2, S1, and S2) were used for statistical analysis. The quality sequences are available from NCBI SRA dataset under the bioproject PRJNA526912 for termite mound soil samples and PRJNA525146 for the surrounding soil samples.

The differences between the physicochemical parameters were determined by one-way analysis of variance (ANOVA) for the comparison of means with Tukey’s pairwise comparison test for significance level (*p*-value < 0.05). Pielou evenness and Shannon diversity indices were assessed for each of the samples and these indices were compared between habitats using a Kruskal–Wallis test. All these analyses were done using PAST version 3.20 [29]. The β diversity was depicted using the principal coordinate analysis (PCoA) based on a Euclidean distance matrix and the one-way analysis of similarities (ANOSIM), via 999 permutations, was used to test for differences in community composition between the groups of samples [30]. The principal component analysis (PCA) based on a Euclidean distance matrix was used to show how these functional categories were distributed between the termite mound and the surrounding soil samples. To find the environmental variables that best explained functional gene composition, we performed canonical correspondence analysis (CCA) and we applied a forward selection of environmental variables and the Monte Carlo permutation test, with 999 random permutations, was used for the significance test. All of the environmental variables listed in Table 2 were included in the CCA analysis as explanatory variables. The PCoA, PCA, and CCA were plotted via CANOCO 5 (Microcomputer Power, Ithaca, NY). The heatmap was drawn using the Shinyheatmap with z-score transformed relative abundance of functional gene categories [31].

## 3. Results

### 3.1. Metagenome Sequencing and Sequence Processing

Examination and annotation of output data were done using the metagenomics rapid annotation online server at http://www.mg-rast.org. The output file after QC contained an average sum of 6,802,220 (T1) and 6,422,685 (T2) retained sequence reads with an average G + C content of 61.25% for the termite mound soil samples, while an average sum of 7,327,766 (S1) and 6,916,304 (S2) sequence reads with an average G + C content of 66.25% were retained for the surrounding soil samples. Of the sequence reads that passed QC, 2,558,335 (T1) and 2,324,880 (T2), the sequence reads from termite mound soils contained predicted proteins with known functions while 2,780,323 (S1) and 2,635,204 (S2) sequence reads from surrounding soil samples contained predicted proteins with known functions. Furthermore, 3,958,147 (T1) and 3,366,939 (T2) sequence reads from termite mound soils contained predicted proteins with unknown functions while 4,258,648 (S1) and 3,985,927 (S1) sequence reads from surrounding soil samples contained predicted proteins with unknown functions.

### 3.2. Functional Analysis Associated with Termite Mound Soils and Their Surrounding Soil Samples

**Figure 2:**
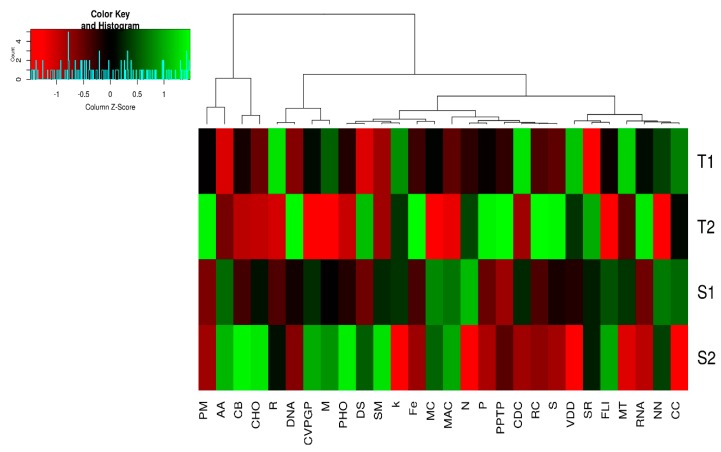
Sequences similar to major metabolisms in termite mound soils and the surrounding soil samples. The scale bar represents color saturation gradient based on the relative abundances with z-score transformed relative abundance of the functional gene categories. Abbreviations are as indicated in the text above.

**Figure 3:**
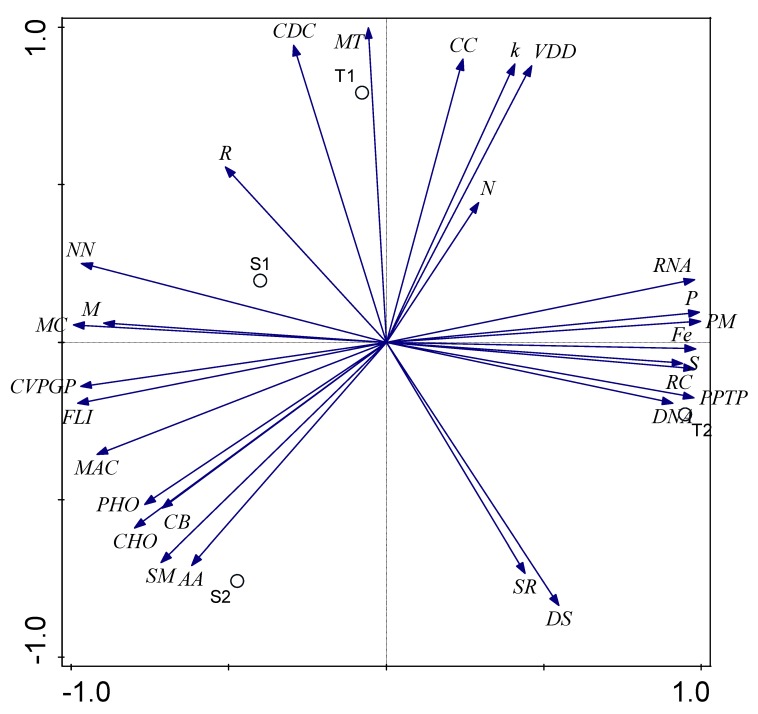
PCA of Functional analysis of bacterial metagenomes. The length of the vectors represents the strength of influence of the particular metabolic process. Axis 1 and axis 2 explained 86.1% and 11.47% variation, respectively.

In this study, at Seed Subsystem level 1 hierarchical gene annotation, twenty-eight (28) major functional categories related to bacteria were found in both termite mound soils and surrounding soil samples, although with different relative abundances. Of the 28 functional categories, 16 differed significantly (*p*-value < 0.05) between the termite mound soils and the surrounding soil samples except for cell division and cell cycle (CDC), cell wall and capsule (CC), clustering-based subsystems (CS), cofactors, vitamins, prosthetic groups, pigments (CVPGP), DNA metabolism (DNA), nitrogen metabolism (N), nucleosides and nucleotides (NN), potassium metabolism (K), respiration (R), secondary metabolism (SM), sulfur metabolism (S), and stress response (SR) (Figure 2 and Table S1). The most predominant functional categories in termite mound soils were iron acquisition and metabolism (Fe), membrane transport (MT), phages, prophages, transposable elements, plasmids (PPTP), phosphorus metabolism (P), protein metabolism (PM), RNA metabolism (RNA), and virulence, disease, and defense (VDD). However, sequences associated with carbohydrate metabolism (CHO), amino acids and derivatives (AA), fatty acids, lipids, and isoprenoids (FLI), metabolism of aromatic compounds (MAC), and motility and chemotaxis (MC) were more abundant in surrounding soil samples (Figure 2). miscellaneous (M), regulation and cell signaling (RC), photosynthesis (PHO), and dormancy and sporulation (DS) were also significantly different (*p*-value < 0.05) between the termite mound soils and the surrounding soil samples. Principal component analysis (PCA) was conducted to show how these functional categories were distributed between the termite mound soils and their surrounding soil samples (Figure 3).

**Figure 4:**
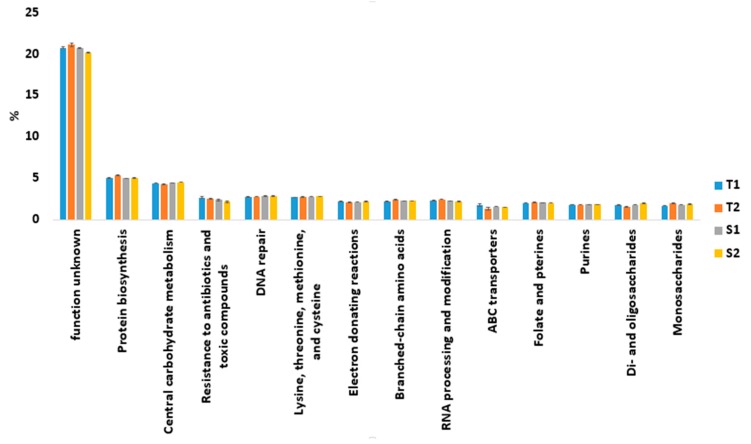
Functional categories based on SEED Subsystem level 2 classification in each soil sample.

At SEED Subsystem level 2 hierarchical gene annotation, the function unknown category was the most abundant across all samples. Their relative abundances were 20.71% (T1) and 21.08% (T2) in termite mound soils and 20.65% (S1) and 20.19% (S2) in surrounding soil samples. This was followed by protein biosynthesis and the relative abundances were 5.01% (T1) and 5.37% (T2) in termite mound soils and 4.98% (S1) and 4.99% (S2) in their surrounding soil samples (Figure 4).

### 3.3. α and β Diversity of the Functional Categories of both Soil Samples

**Figure 5:**
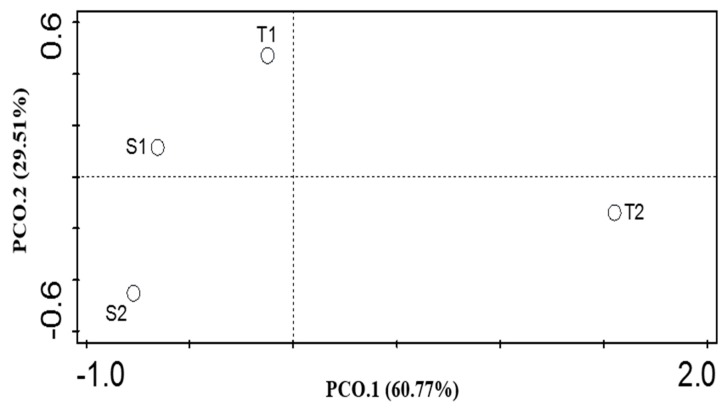
Principal coordinate analysis (PCoA) for functional categories at Subsystem level 1 obtained from termite mound soils and surrounding soil samples.

The functional diversity at SEED Subsystem level 1, measured using the Shannon index and the evenness index, did not differ significantly (*p* > 0.05) among the termite mound soils and the surrounding soil samples (Table 1). A Kruskal–Wallis test revealed that the level of diversity difference between the soil samples from the mounds was not significant (*p* = 0.97) while the level of diversity difference between the surrounding soil samples was also not significant (*p* = 0.92). The principal coordinate analysis (PCoA) plot was used to visualize the samples based on relative abundances of annotations within Subsystems level 1 (Figure 5). The analysis of similarity (ANOSIM) showed that *p* = 0.01, *R* = 0.58.

### 3.4. Physiochemical Characterization of the Termite Mound Soils and Their Surrounding Soil Samples

From the soil analysis, it was observed that the soil pH levels (T1 = 5.10 and T2 = 4.48) in termite mound soils were significantly (*p*-value < 0.05) more acidic than the pH (S1 = 5.80 and S2 = 5.38) in their corresponding surrounding soil samples. The sand, clay, K, Ca, and N contents differed significantly (*p*-value < 0.05) among both habitats. The clay, Ca, and K contents were all significantly (*p*-value < 0.05) higher in termite mound soils than the surrounding soil samples, while the sand and N contents were significantly (*p*-value < 0.05) higher in surrounding soil samples than the termite mound soils (Table 2).

### 3.5. Influence of Environmental Factors on Bacterial Functional Category

**Figure 6:**
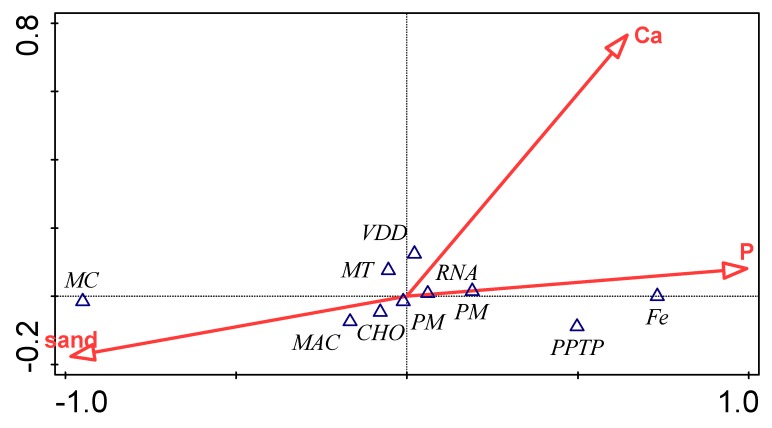
Canonical correspondence analysis (CCA) of functional categories and major soil chemical parameters for both samples.

The relationship between the measured soil parameters and the relative abundances within bacterial functional categories at Subsystem level 1 were investigated using canonical correspondence analysis. Three parameters, including sand, P, and Ca, were selected for CCA based on the significant test (Figure 6) and, as shown in Table 3, the environmental factor that best explains variation in functional gene composition.

## 4. Discussion

Evaluating bacterial functional genes that take part in key biogeochemical processes is essential in relating bacterial community structures to their potential ecological functions [18]. The SEED assembles metabolic pathways into a hierarchical structure where by the total genes essential for a particular assignment are organized into subsystems. At the highest level of organization, the subsystems include both anabolic and catabolic functions and at the lowest levels it includes specific pathways [32].

Our α diversity result showed that the functional diversity represented by the metagenomes in both termite mound and surrounding soils approached its theoretical limit of 2.81 [33], showing that most subsystems were represented in all of the samples. The evenness for the metagenomes was low (around 0.61, Table 1), showing that there are a few dominant metabolisms (like carbohydrate and amino acids and derivatives) in each environment. Differential dominant metabolisms suggest that there are characteristic functional profiles of the metagenomes.

The bacterial functional categories did not differ significantly (*p*-value > 0.05) within the habitats (Table 1). However, there were clear separations (the strength of the separation (R) = 0.58) between the termite mound soils and the surrounding soil samples from our PCoA and 90.28% of the combined PCoA axis 1 and 2 explained the community variation (Figure 5). This was tested with the analysis of similarity (ANOSIM), which showed that the bacteria functional categories in termite mound soils and their comparative surrounding soils varied significantly (*p*-value = 0.01).

To test the hypothesis that each environment has a distinguishing metabolic profile, a principal component analysis (PCA) was conducted (Figure 3). Most of the variance between the termite mound soils and the surrounding soil samples (c. 97.57%) was explained in this analysis, showing that metagenomes are highly predictive of metabolic potential within an ecosystem. The position of each metagenome in Figure 2 reflects the frequency combination of sequences associated with each subsystem; the vector arrows show which metabolisms most strongly determined the distribution. Using these as clues, it is possible to determine which metabolisms are important for the bacteria in each environment relative to other environments. For example, subsystems involved in iron acquisition and metabolism, phages, prophages, transposable elements, plasmids, phosphorus metabolism, protein metabolism, RNA metabolism, regulation and cell signaling, RNA metabolism, and sulfur metabolism placed the termite mound soil bacteria (T2) apart from the bacteria found within S1, S2, and T1.

From our results it was observed that each environment has a dominant functional gene category. The predominant of sequences associated with carbohydrate metabolism, amino acids and derivatives, fatty acids, lipids, and isoprenoids, metabolism of aromatic compounds, and motility and chemotaxis in the surrounding soil samples were expected (Figure 2 and Supplementary Table S1). This is because bacteria depend on the bioavailability of carbon, which is a major factor for their growth and metabolism [34]. This became evident with most of the sequences related to central carbohydrate metabolism, disaccharides, and oligosaccharides (Figure 4) and the large number of metabolic pathways involved in the carbon cycle, like the serine glyoxylate cycle, were most abundant across S1 and S2 samples (Supplementary Figure S2A). Gianoulis et al. [35] explained that bacteria mostly utilize amino acids as sources of energy in areas with limited plant nutrients and organic matter. Dhembare [10] and Deke, Adugna, and Fite [11] have previously revealed that termite mound soils were richer in plant nutrients such as calcium, phosphorus, magnesium, potassium, and organic matter, than their surrounding soils. This became clear from our soil analysis, which showed that termite mound soils were richer in phosphorus, calcium, magnesium, and potassium than the surrounding soil samples (Table 2). With these low nutrient concentrations in the surrounding soils [36], it is likely that high abundances of competitive related genes, such as motility and chemotaxis, will be advantageous for bacteria thriving in such habitats, as it would help them to move, communicate, and rapidly acquire available nutrients.

As expected, our study also revealed the predominant of sequences related to iron acquisition and metabolism, virulence, disease and defense, phages, prophages, transposable elements, and housekeeping genes (such as RNA metabolism and protein metabolism) in termite mound soils. These functional genes are needed in order to provide meaningful protection to the termite’s colony against fungal entomopathogens [20]. Sequences related to nitrogen, sulfur, and phosphorus metabolism, which are linked with nutrient cycling, were recorded in both termite mound soils and the surrounding soil samples, though their relative abundance did not differ significantly (*p*-value > 0.05), except for phosphorus metabolism. Soil bacteria are key drivers of soil nitrogen, sulfur, and the phosphorus cycle [37] and this was confirmed with the presence of high relative abundances of metabolic processes in nitrogen cycling (such as ammonia assimilation, nitrate and nitrite ammonification, nitric oxide synthase, allantoin utilization, denitrification, and nitrogen fixation in both types of samples (Figure S2C)), metabolic processes related to alkanesulfonate assimilation, sulfur oxidation, utilization of glutathione, inorganic sulfur assimilation, galactosylceramide and sulfatide metabolism (Figure S2B), and phosphate metabolism (Supplementary Figure S2D). Clustering based on subsystems, which have been defined as functionally coupled genes with unknown functions by Castañeda and Barbosa [38] and Uroz et al. [39], were the second most abundant functional category in this study. With the high proportion of clustering-based on subsystems in the entire functional categories and the function unknown in Subsystem level 2 (Figure 2 and Figure 4 respectively), it shows the level of bacteria genes present in soil whose functionalities are largely underexploited.

We also hypothesized that bacterial functional diversity will be driven by soil physicochemical parameters. The canonical correspondence analyses (CCA) plot (Figure 6) indicated the functional categories are likely dependent on the soil properties. The vector length of Ca (on the axis 1) positively correlated with phosphorus metabolism, protein metabolism, membrane transport, virulence, disease, and defense. On axis 2, the vector length of P positively correlated with carbohydrates, RNA metabolism, iron acquisition and metabolism, and phages, prophages, transposable elements, plasmids, but negatively correlated with motility and chemotaxis. The CCA result of the Subsystem level 1 functional category showed that P was the best predictor for functional category composition, alone explaining 92.8% of the total variation (Table 3, Figure 6). Environmental factors have been acknowledged as the main drivers of soil bacterial distribution with physicochemical parameters, seen as a principal variable elucidating larger quotas of the variations in soil bacterial diversity and structure [40,41]. Soil physical and chemical parameters have been reported to influence bacterial functional categories [42]. This showed then that soil physicochemical parameters in this study, influenced the relative abundance of bacteria functional categories in both environments.

## 5. Conclusions

This study, to our knowledge, represents a foremost effort to unveil the functional profile of bacteria in termite mound soils using a shotgun metagenomic approach. Our metagenomic analysis showed that termite mound soils and their surrounding soil samples housed similar bacteria functional categories, of which the relative abundances of 16 functional categories differed significantly between both habitats. Furthermore, our α diversity analysis showed no significant difference in bacterial functional categories within both habitats, while the β diversity showed that bacterial functional categories varied significantly between the termite mound soils and the surrounding soil samples. We also reported the influence of physicochemical parameters on the relative abundances of the functional categories in both environments. This study also revealed high relative abundances of functional categories with unknown functions, signifying the likelihood of getting novel genes from the metagenome. We therefore recommend more studies to further investigate the functional gene categories in termite mound soils. This could help to unveil the ecological roles of these function unknown categories, which may play a vital part in the biogeochemical cycle and industrial and biotechnological processes.

## Figures and Tables

**Table 1:** Diversity and evenness estimation of the functional categories of soil samples at SEED Subsystem level 1.

	T1	T2	S1	S2	*p*-Value
Shannon_H	2.86 ± 0.16	2.87 ± 0.16	2.85 ± 0.16	2.84 ± 0.16	0.99
Evenness_e^H/S	0.62 ± 0.06	0.63 ± 0.06	0.62 ± 0.07	0.61 ± 0.07	

Mean ± standard deviation (*n* = 4). *p*-values based on Kruskal–Wallis test.

**Table 2:** Soil analysis of termite mound soils and their comparative surrounding soils.

Soil Property	T1	T2	S1	S2
Sand (%)	65.00 ± 8.29a	47.75 ± 23.60b	72.00 ± 17.66c	76.50 ± 3.00d
Silt (%)	9.00 ± 2.94a	19.75 ± 11.38a	11.75 ± 12.87a	10.25 ± 0.96a
Clay (%)	26.00 ± 6.27a	33.25 ± 13.52a	16.25 ± 4.50b	13.25 ± 3.20c
K (mg/L)	393.50 ± 120.33a	427.50 ± 57.93a	216.75 ± 48.40b	184.50 ± 27.72c
Ca (mg/L)	1879.50 ± 587.38a	2237.75 ± 318.91a	1493.50 ± 456.59a	1108.50 ± 160.48b
Mg (mg/L)	575.00 ± 262.32a	622.25 ± 60.84a	349.75 ± 159.70a	330.25 ± 138.75a
pH	5.10 ± 0.33a	4.48 ± 0.46a	5.80 ± 0.32b	5.38 ± 0.39c
N (%)	0.09 ± 0.03a	0.10 ± 0.03b	0.59 ± 0.47c	0.25 ± 0.04d
P (mg/L)	0.25 ± 0.50a	0.75 ± 0.50a	0.00 ± 0.00a	0.00 ± 0.00a
OC (%)	0.31 ± 0.42a	0.10 ± 0.00a	0.11 ± 0.0a	0.11 ± 0.01a

Mean ± standard deviation (*n* = 4). Mean values in a same row with different letters (a, b, c, and d) were significantly different (*p*-value < 0.05) based on Tukey’s pairwise significant difference test.

**Table 3:** Forward selection of environmental variables, which best explain variation in functional gene composition (Subsystem level 1 genes) between samples.

Environmental Variable	Explains %	Contribution %	Pseudo-F	*P*
P	92.8	92.8	25.8	0.09
Ca	5.1	5.1	2.4	0.87
Sand	2.1	2.1	<0.1	1.

## Supplementary Materials

The following are available online at https://www.mdpi.com/2073-4425/10/9/637/s1, Figure S1: Heatmap of bacterial phyla in termite mound soils and their corresponding surrounding soil samples, Figure S2A: relative abundance of pathways involved in carbohydrate metabolism in both soil samples, Figure S2B: relative abundance of pathways involved in sulphur metabolism in both samples, Figure S2C: relative abundance of pathways involved in nitrogen metabolism in both samples, Figure S2D: relative abundance of pathways involved in phosphorus metabolism in both samples, Table S1: Mean percentage of sequences similar to major metabolisms in termite mound soils and the surrounding soils.
