# Supplementary material for: Profiling the Functional Diversity of Termite Mound Soil Bacteria as Revealed by Shotgun Sequencing
Source: Genes (Basel). 2019 Aug 23;10(9):637. doi: 10.3390/genes10090637 (PMC6770954; doi:10.3390/genes10090637)
Supplement: Supplementary file 1 [file genes-10-00637-s001.pdf]

Profiling the functional diversity of termite mound soil bacteria as revealed by shotgun sequencing

Ben Jesuorsemwen Enagbonma, Bukola Rhoda Aremu and Olubukola Oluranti Babalola

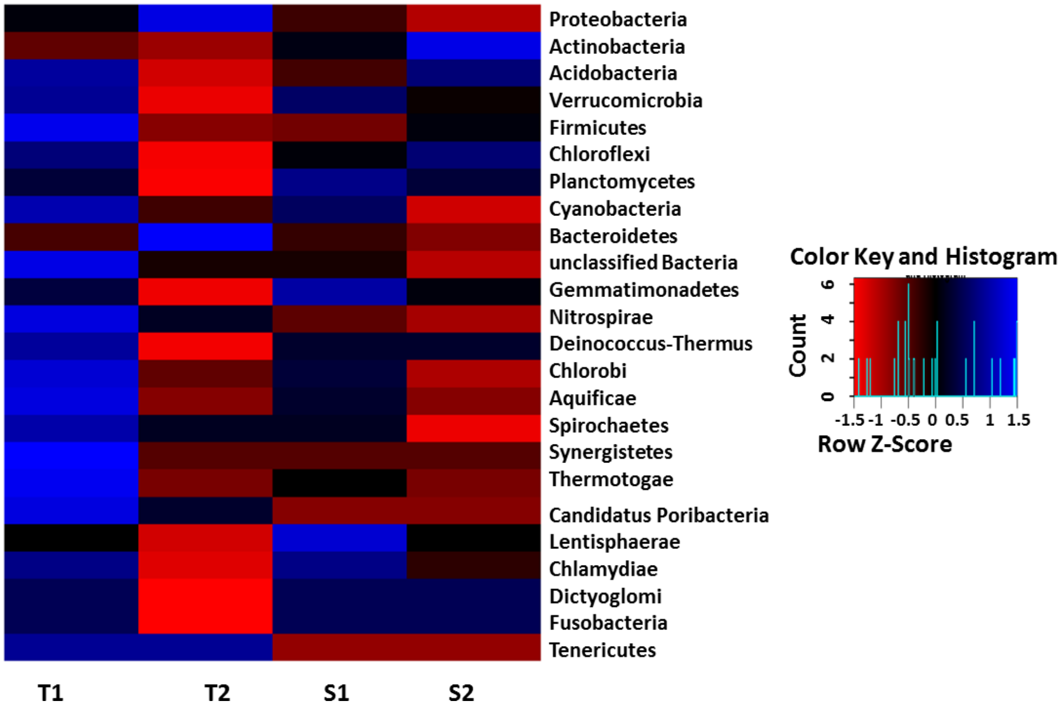

Figure S1: Heatmap of bacterial phyla in termite mound soils and their corresponding surrounding soil samples.

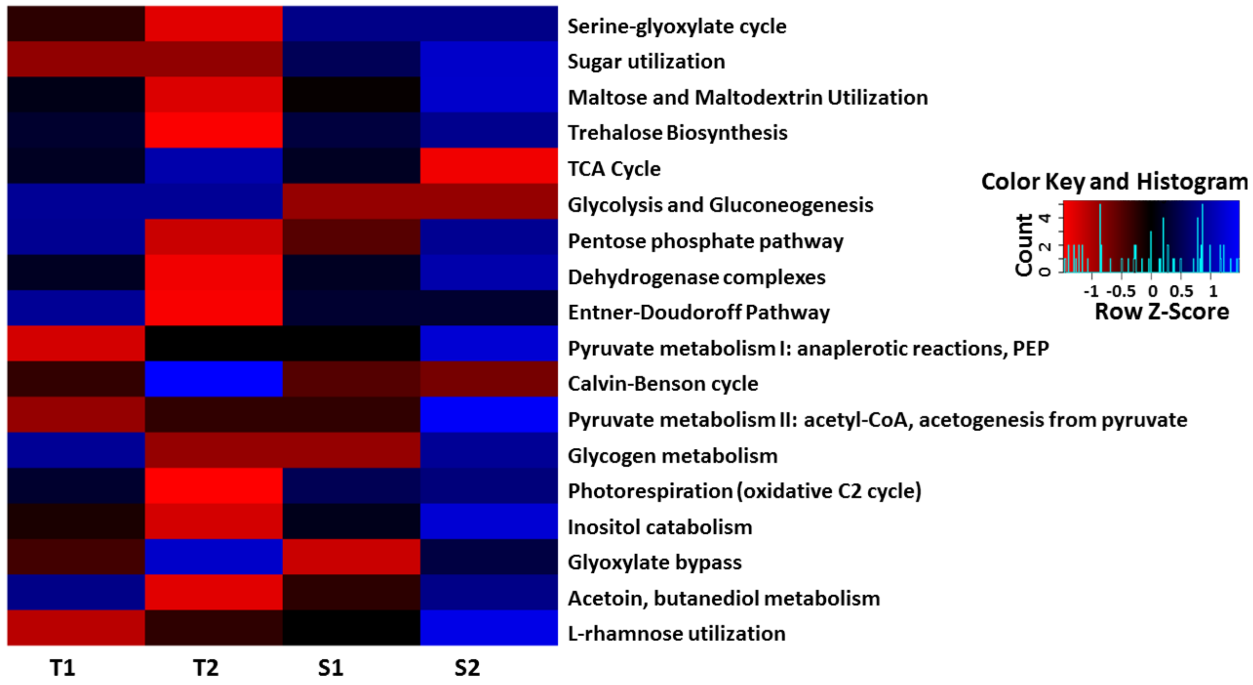

Figure S2A: relative abundance of pathways involved in carbohydrate metabolism in both soil samples.

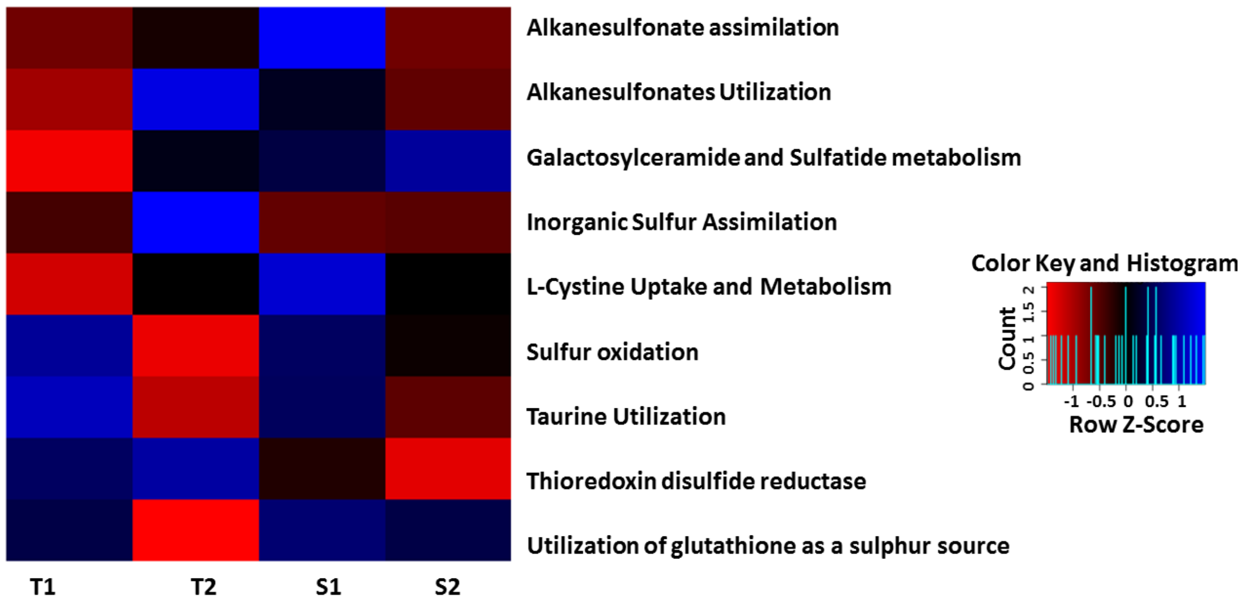

Figure S2B: relative abundance of pathways involved in sulphur metabolism in both samples.

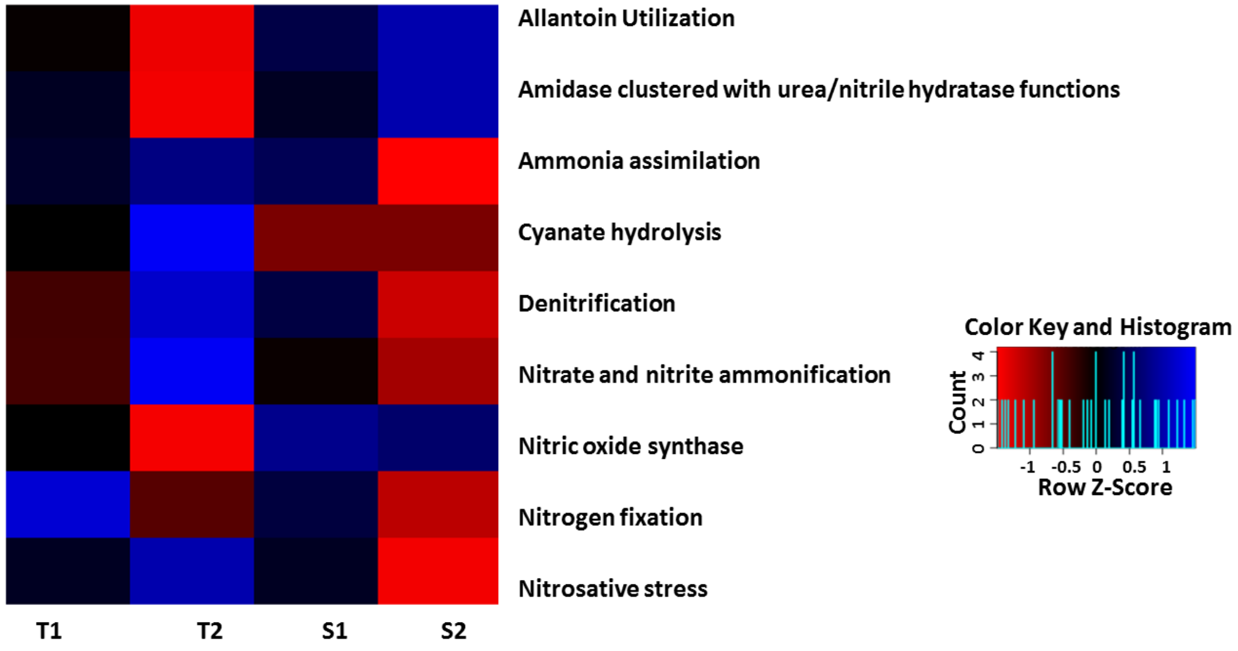

Figure S2C: relative abundance of pathways involved in nitrogen metabolism in both samples.

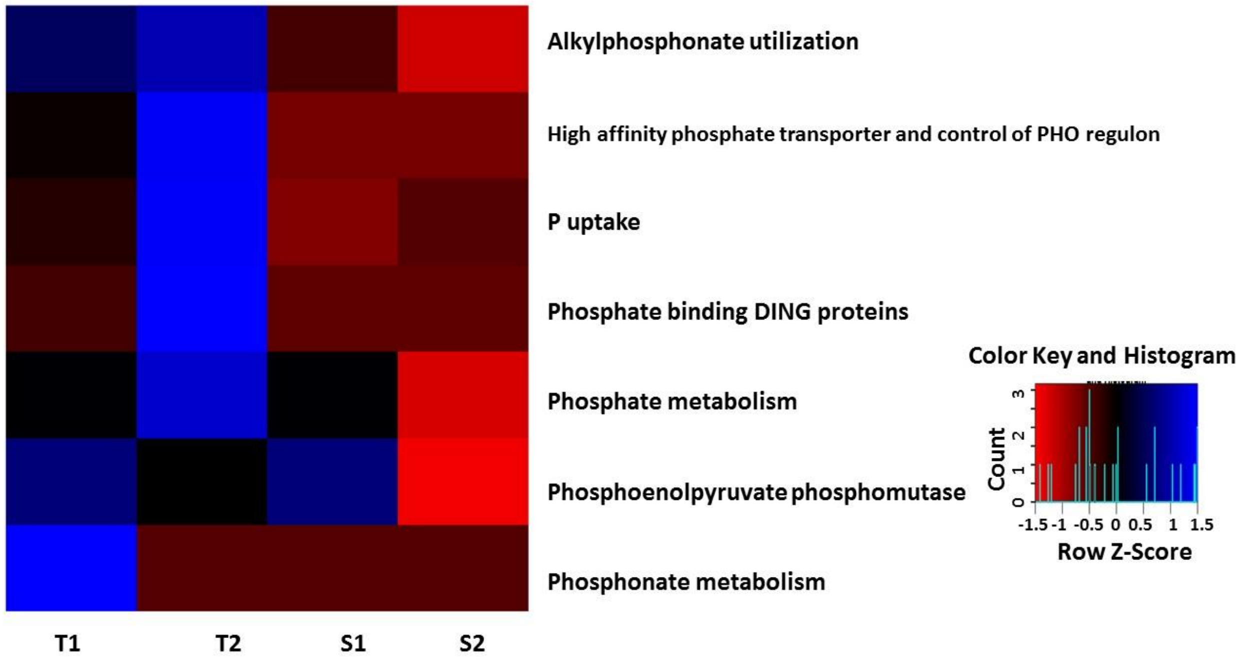

Figure S2D: relative abundance of pathways involved in phosphorus metabolism in both samples

Table S1: Mean percentage of sequences similar to major metabolisms in termite mound soils and the surrounding soils

Functional categories based SEED level 1	T1	T2	S1	S2	P-value
Amino Acids and Derivatives	10.31 ± 0.16a	10.38 ± 0.04a	10.55 ± 0.19a	10.61 ± 0.10b	0.03
Carbohydrates	13.93 ± 0.23a	13.72 ± 0.26a	14.25 ± 0.16b	14.82 ± 0.27c	0.01
Cell Division and Cell Cycle	0.94 ± 0.03a	0.92 ± 0.03a	0.93 ± 0.04a	0.92 ± 0.01a	0.73
Cell Wall and Capsule	3.49 ± 0.08a	3.44 ± 0.17a	3.48 ± 0.12a	3.32 ± 0.03a	0.12
Clustering-based subsystems	13.13 ± 0.10a	13.09 ± 0.03a	13.12 ± 0.10a	13.21 ± 0.09a	0.24
Cofactors, Vitamins, Prosthetic Groups, Pigments	5.98 ± 0.12a	5.81 ± 0.22a	6.00 ± 0.07a	6.08 ± 0.10a	0.28
DNA Metabolism	4.50 ± 0.13a	4.66 ± 0.14a	4.55 ± 0.10a	4.50 ± 0.12a	0.46
Dormancy and Sporulation	0.14 ± 0.01a	0.18 ± 0.02b	0.15 ± 0.01a	0.17 ± 0.03a	0.03
Fatty Acids, Lipids, and Isoprenoids	2.65 ± 0.07a	2.52 ± 0.09a	2.71 ± 0.03b	2.76 ± 0.04c	0.01
Iron acquisition and metabolism	0.59 ± 0.11a	0.91 ± 0.17b	0.58 ± 0.05c	0.51 ± 0.04d	0.02
Membrane Transport	3.89 ± 0.27a	3.59 ± 0.21a	3.73 ± 0.08a	3.47 ± 0.03b	0.01
Metabolism of Aromatic Compounds	1.76 ± 0.07a	1.69 ± 0.10a	1.88 ± 0.08b	1.91 ± 0.02c	0.01
Miscellaneous	6.51 ± 0.03a	6.31 ± 0.03b	6.45 ± 0.08c	6.54 ± 0.08d	0.02
Motility and Chemotaxis	0.71 ± 0.08a	0.41 ± 0.06b	0.86 ± 0.11c	0.82 ± 0.07d	0.01
Nitrogen Metabolism	1.29 ± 0.78a	1.31 ± 0.06a	1.33 ± 0.08a	1.26 ± 0.03a	0.39
Nucleosides and Nucleotides	3.35 ± 0.02a	3.23 ± 0.15a	3.37 ± 0.04a	3.35 ± 0.03a	0.45
Phages, Prophages, Transposable elements, Plasmids	0.97 ± 0.04a	1.34 ± 0.28b	0.86 ± 0.04b	0.93 ± 0.03d	0.01
Phosphorus Metabolism	1.34 ± 0.02a	1.53 ± 0.05b	1.28 ± 0.06c	1.24 ± 0.02d	0.01
Photosynthesis	0.09 ± 0.01a	0.08 ± 0.01a	0.09 ± 0.01b	0.11 ± 0.01c	0.01
Potassium metabolism	0.47 ± 0.04a	0.45 ± 0.04a	0.45 ± 0.05a	0.38 ± 0.03a	0.09
Protein Metabolism	8.32 ± 0.11a	8.59 ± 0.12b	8.21 ± 0.13b	8.18 ± 0.08c	0.01
RNA Metabolism	3.47 ± 0.10a	3.71 ± 0.03b	3.34 ± 0.09c	3.27 ± 0.04d	0.01
Regulation and Cell signaling	0.93 ± 0.02a	1.10 ± 0.06b	0.93 ± 0.01c	0.90 ± 0.01d	0.01
Respiration	4.55 ± 0.14a	4.33 ± 0.06a	4.39 ± 0.02a	4.44 ± 0.12a	0.17
Secondary Metabolism	0.30 ± 0.01a	0.30 ± 0.01a	0.31 ± 0.01a	0.32 ± 0.01a	0.07
Stress Response	2.40 ± 0.06a	2.52 ± 0.08a	2.48 ± 0.08a	2.48 ± 0.04a	0.12
Sulfur Metabolism	0.98 ± 0.04a	1.04 ± 0.05a	0.99 ± 0.04a	0.97 ± 0.04a	0.24
Virulence, Disease and Defense	3.04 ± 0.20a	2.87 ± 0.16a	2.76 ± 0.17a	2.54 ± 0.17b	0.04

A) Mean ± standard deviation (n= 4) .b) Different letters in a same row denote significant differences ($P < 0.05$) by Tukey's pairwise significant difference test.
